# Neonatal bronchopulmonary dysplasia severity and respiratory outcomes at 3 years of age: a prospective cohort study

**DOI:** 10.3389/fped.2026.1881107

**Published:** 2026-07-30

**Authors:** Jiajia Ying, Jingqian Zhou, Hongfang Chen, Jian Zhou

**Affiliations:** Department of Pediatrics, The First People’s Hospital of Yongkang, Jinhua, Zhejiang, China

**Keywords:** asthma, bronchopulmonary dysplasia, longitudinal follow-up, modified severity category, preterm infant, pulmonary function

## Abstract

**Objective:**

To examine whether bronchopulmonary dysplasia (BPD) severity is associated with pulmonary function abnormalities and asthma diagnosis risk at 3 years of age, and to support risk-stratified respiratory follow-up in children with BPD.

**Methods:**

This prospective cohort study included 68 preterm infants admitted to the neonatal intensive care unit of The First People's Hospital of Yongkang between January 2022 and December 2022. In the study protocol, BPD was operationally defined at 36 weeks' postmenstrual age as persistent supplemental oxygen requirement [fraction of inspired oxygen (FiO2) > 0.21] to maintain target oxygen saturation of 90%–95%, together with persistent pulmonary parenchymal abnormalities on chest imaging. Severity was then categorized using a prespecified support- and oxygen-based clinical scheme informed by, but not identical to, the 2018 National Institute of Child Health and Human Development (NICHD) workshop framework. Participants were followed until 3 years of age, when conventional spirometry and guideline-based clinical asthma assessment were performed. The associations of modified BPD grade with pulmonary function indices, asthma diagnosis, and asthma clinical risk categories were analyzed.

**Results:**

Among the 68 infants with BPD, 29 (42.6%) had grade I BPD, 23 (33.8%) had grade II BPD, and 16 (23.5%) had grade III BPD. Sixty children completed both pulmonary function testing and asthma assessment at 3 years of age, yielding a follow-up rate of 88.2%. Percent predicted forced expiratory volume in 1 s (FEV1), FEV1/forced vital capacity (FVC), and maximal mid-expiratory flow (MMEF) were significantly lower in the grade III BPD group than in the grade I and grade II groups (all *P* < 0.01). The asthma diagnosis rate was significantly higher in the grade III group (46.7%) than in the grade I (8.0%) and grade II (20.0%) groups (chi-square = 8.156, *P* = 0.017). In an exploratory multivariable logistic regression model, grade III BPD [odds ratio [OR], 5.72; 95% confidence interval [CI], 1.89–17.31; *P* = 0.002] and birth weight <1,000 g (OR, 3.46; 95% CI, 1.12–10.68; *P* = 0.031) were associated with asthma diagnosis at 3 years of age.

**Conclusion:**

Neonatal BPD severity was associated with the degree of pulmonary function impairment and with asthma diagnosis risk at 3 years of age. Children with grade III BPD had poorer long-term respiratory outcomes and should be considered a higher-risk group requiring structured longitudinal respiratory follow-up. Because this was a selected survivor cohort with structured follow-up, and because asthma diagnosis in preschool children with BPD can overlap with post-prematurity airway disease, these findings should be interpreted as risk-marker associations rather than causal evidence.

## Introduction

Bronchopulmonary dysplasia (BPD) is one of the most common chronic respiratory complications of preterm birth. Contemporary BPD is characterized by arrested alveolarization, dysregulated pulmonary microvascular development, and persistent impairment of lung growth and airway function (1–4). Advances in perinatal and neonatal intensive care have substantially improved the survival of very-low-birth-weight and extremely-low-birth-weight infants; however, BPD remains a major source of morbidity among extremely preterm survivors (5–7). Recent reviews have also emphasized that BPD should be considered within the broader spectrum of prematurity-associated lung disease and long-term post-prematurity respiratory sequelae (8–11).

The clinical significance of BPD extends beyond the neonatal period. Children and adolescents born preterm, particularly those with a history of BPD, have lower lung function, more frequent respiratory symptoms, and a higher risk of obstructive airway disease throughout childhood and early adulthood than term-born peers (12–20). Systematic reviews and meta-analyses have consistently shown reductions in forced expiratory volume in 1 s (FEV1) among preterm-born survivors, with larger deficits among those who had BPD in infancy (16–20). Nevertheless, evidence linking neonatal BPD severity to preschool pulmonary function and asthma diagnosis risk remains limited, particularly in longitudinal cohorts from China.

The 2018 National Institute of Child Health and Human Development (NICHD) workshop proposed a revised approach to BPD definition and severity grading that incorporates respiratory support requirements and radiographic evidence at 36 weeks' postmenstrual age (3). Subsequent studies have examined the prognostic performance of newer BPD definitions for early childhood morbidity and longer-term respiratory outcomes, but findings remain heterogeneous (4–7). Further clinical validation is therefore needed to determine whether these severity categories can identify children at increased risk of preschool respiratory morbidity.

Asthma is one of the most common chronic respiratory diseases in childhood. Both BPD-related airway disease and asthma may present with recurrent wheeze, cough, and exercise intolerance, but their underlying mechanisms are not identical. BPD-related wheeze is largely driven by impaired lung development, airway remodeling, and structural airway dysfunction, whereas classic childhood asthma is usually characterized by variable airflow limitation and airway inflammation (21–26). Previous meta-analytic evidence indicates that preterm infants with BPD have an increased risk of subsequent asthma or asthma-like disease (24–26). Clarifying the predictive value of BPD severity for asthma diagnosis at 3 years of age could support risk-stratified surveillance and earlier respiratory intervention.

On this basis, the present prospective cohort study enrolled preterm infants with BPD admitted to the neonatal intensive care unit of The First People's Hospital of Yongkang. BPD was identified using a prespecified operational definition, and severity was categorized at 36 weeks' postmenstrual age using a modified clinical scheme based on respiratory support and oxygen requirement. Children were followed to 3 years of age to evaluate pulmonary function, asthma diagnosis, and asthma risk categories. We aimed to determine whether this neonatal BPD severity category can serve as a clinically useful risk marker for preschool pulmonary function impairment and asthma diagnosis risk.

## Materials and methods

### Study population

This prospective cohort study included preterm infants admitted to the neonatal intensive care unit of The First People's Hospital of Yongkang between January 2022 and December 2022. The inclusion criteria were as follows: (1) gestational age <32 weeks; (2) diagnosis of BPD at 36 weeks' postmenstrual age according to the prespecified operational study definition described below; and (3) written informed consent from a parent or legal guardian.

Infants were excluded if they had congenital pulmonary hypoplasia or pulmonary sequestration; complex congenital heart disease other than isolated patent ductus arteriosus or small atrial/ventricular septal defects; congenital airway malformations such as tracheoesophageal fistula, laryngeal cleft, or tracheomalacia; or chromosomal abnormalities or inherited metabolic disorders. Because BPD diagnosis and modified severity categorization were performed at 36 weeks' postmenstrual age, infants who died before this assessment were not eligible for this BPD cohort. Severe neurodevelopmental impairment was not used to remove children from the neonatal BPD cohort after enrollment. If a child was unable to complete acceptable spirometry at 3 years of age, the pulmonary function outcome was treated as unavailable rather than as a *post hoc* exclusion from the cohort.

The local NICU clinical management context is summarized in Supplementary Methods. In brief, respiratory care was delivered according to local protocols based on Chinese neonatal consensus recommendations, with oxygen titrated to a target saturation range of 90%–95% and respiratory support adjusted according to clinical stability, blood gas results, apnea burden, and work of breathing.

### Operational BPD definition and severity categorization

The study protocol specified both an operational BPD case definition and a severity categorization before assessment of 3-year respiratory outcomes. At 36 weeks' postmenstrual age, BPD was operationally defined as a persistent need for supplemental oxygen (FiO2 > 0.21) to maintain a target oxygen saturation of 90%–95%, together with persistent pulmonary parenchymal abnormalities on chest imaging judged compatible with BPD by the neonatal clinical team. This operational definition was applied consistently to identify the neonatal BPD cohort.

For severity categorization, infants meeting this operational BPD definition were classified using a prespecified modified clinical grading scheme informed by the 2018 NICHD workshop framework (3). This scheme was not a direct implementation of the published NICHD or Jensen grading systems. It was chosen because respiratory support mode, FiO2, and chest imaging status were uniformly available in the local electronic medical record, whereas some flow-based support details required for exact application of the NICHD or Jensen definitions were not consistently recorded across the full cohort. Applying those definitions retrospectively would therefore have introduced missingness and potential misclassification. The modified scheme was used as a pragmatic clinical severity marker for internal risk stratification, not as a replacement for internationally accepted BPD definitions.

Each infant was assigned to the highest applicable grade according to a hierarchical rule based on respiratory support intensity. Grade I BPD was defined as no positive-pressure respiratory support and either room air with persistent radiographic evidence of pulmonary parenchymal disease or nasal cannula oxygen with FiO2 ≤0.30. Grade II BPD was defined as nasal cannula oxygen, nasal continuous positive airway pressure, or nasal intermittent positive-pressure ventilation with FiO2 > 0.21 and <0.30 in the absence of invasive mechanical ventilation. Grade III BPD was defined as invasive mechanical ventilation at any FiO2, or nasal continuous positive airway pressure or nasal intermittent positive-pressure ventilation with FiO2 ≥ 0.30. Thus, infants receiving invasive mechanical ventilation with FiO2 < 0.30, if present, were classified as grade III on the basis of respiratory support mode.

### Clinical data collection

Perinatal and neonatal data were extracted from the electronic medical record system. Variables included sex, gestational age, birth weight, mode of delivery, 5 min Apgar score, antenatal corticosteroid exposure, respiratory distress syndrome (RDS), surfactant therapy, patent ductus arteriosus (PDA), duration of invasive mechanical ventilation, length of hospitalization, intraventricular hemorrhage (IVH; grade II or higher), retinopathy of prematurity (ROP), and nosocomial infection. The Clinical Risk Index for Babies II (CRIB-II) score was not routinely recorded in the electronic medical record during the study period and was therefore unavailable for analysis.

### Follow-up protocol

After discharge, all infants with BPD were enrolled in a structured disease-specific follow-up program. Outpatient follow-up visits were scheduled at corrected ages of 1, 3, 6, 12, 24, and 36 months. Follow-up assessments included growth and developmental evaluation, respiratory symptom surveillance, recording of wheeze, cough, nocturnal awakening, exercise tolerance, respiratory tract infection episodes, and medication use. This follow-up structure may have increased family engagement and completion of the 3-year visit compared with less structured care pathways.

### Pulmonary function testing

Conventional spirometry was performed at 3 years of age. Short-acting bronchodilators were withheld for at least 8 h and long-acting bronchodilators for at least 24 h before testing. Spirometry was performed with a MasterScreen pulmonary function system (Jaeger, Germany) by trained technicians using standardized operating procedures and quality-control criteria for pediatric pulmonary function testing (27–31). Child-friendly coaching and repeated demonstrations were used before and during testing. Each child was asked to complete at least three acceptable maneuvers, and the best value was used for analysis.

The main pulmonary function indices were FEV1 expressed as a percentage of the predicted value (FEV1% predicted), FVC expressed as a percentage of the predicted value (FVC% predicted), FEV1/FVC expressed as a percentage of the predicted value, and MMEF expressed as a percentage of the predicted value (MMEF% predicted). Obstructive ventilatory impairment was defined as FEV1/FVC <92% of the predicted value. Restrictive ventilatory impairment was defined as FVC <80% of the predicted value. Mixed ventilatory impairment was defined as fulfillment of both criteria.

### Asthma diagnosis and clinical risk classification

Asthma diagnosis and clinical risk classification were based on the Chinese Guideline for the Diagnosis and Prevention of Bronchial Asthma in Children and the Global Initiative for Asthma recommendations for young children (32–35). Because asthma diagnosis before 5 years of age is primarily clinical, the diagnosis required recurrent wheeze, cough, dyspnea, or chest tightness plus supportive clinical evidence. Supportive evidence included symptoms triggered by upper respiratory tract infection, exercise, cold-air exposure, or allergen exposure; wheezing on auscultation during symptomatic episodes; a positive bronchodilator response when reliable pre- and post-bronchodilator testing was feasible; repeated clinical response to anti-asthma therapy; and exclusion of other causes of recurrent wheeze. Family history of atopy, eczema, allergic rhinitis, and allergen sensitization were reviewed when available, but these data were not systematically collected and were not included as covariates.

Asthma clinical risk was classified according to symptom burden and treatment requirement. Low risk was defined as diagnosed asthma or recurrent wheezing symptoms that did not require long-term controller therapy. Moderate risk was defined as diagnosed asthma requiring low-dose inhaled corticosteroid (ICS) therapy. High risk was defined as diagnosed asthma requiring medium- or high-dose ICS, ICS plus a long-acting beta2-agonist, or at least two acute exacerbations requiring systemic corticosteroids during the preceding year.

### Statistical analysis

Statistical analyses were performed using SPSS version 26.0 (IBM Corp, Armonk, NY, USA). Continuous variables are presented as mean ± standard deviation and were compared among groups using one-way analysis of variance. Pairwise comparisons were performed using the least significant difference method. Categorical variables are presented as number and percentage and were compared using the chi-square test or Fisher exact test, as appropriate. Trends across ordered modified BPD categories were evaluated using the Cochran-Armitage trend test. Spearman rank correlation analysis was used to assess associations between modified BPD grade and pulmonary function indices.

Candidate predictors for asthma diagnosis were selected based on biological plausibility and prior evidence linking prematurity, neonatal respiratory morbidity, and post-prematurity respiratory outcomes (16–20, 24–26, 36–38). Candidate variables included modified BPD grade, gestational age, birth weight <1,000 g, duration of invasive mechanical ventilation, RDS, PDA, and nosocomial infection. Univariable binary logistic regression was first performed. To limit overfitting in view of the small number of asthma events, variables with *P* < 0.10 in univariable analysis were then entered into an exploratory multivariable binary logistic regression model using the enter method. Duration of invasive mechanical ventilation was analyzed per 1-day increase. Multicollinearity was evaluated using pairwise correlations and variance inflation factors; variables with substantial conceptual and statistical collinearity were not entered simultaneously if this destabilized model estimation. Model calibration was assessed using the Hosmer-Lemeshow goodness-of-fit test, but all model diagnostics were interpreted cautiously because only 13 asthma events were available. A two-sided *P* value <0.05 was considered statistically significant.

## Results

### Baseline clinical characteristics

A total of 68 infants with BPD were included after BPD diagnosis and severity grading at 36 weeks' postmenstrual age. Of these, 42 (61.8%) were male and 26 (38.2%) were female. The mean gestational age at birth was 29.1 ± 2.0 weeks (range, 24 to 31 + 6 weeks), and the mean birth weight was 1,186 ± 268 g (range, 680–1,490 g). According to the modified support- and oxygen-based BPD severity scheme, 29 infants (42.6%) were classified as grade I, 23 (33.8%) as grade II, and 16 (23.5%) as grade III. No enrolled child was excluded from the cohort solely because of severe neurodevelopmental impairment after BPD classification, but formal Bayley-III neurodevelopmental testing was not performed concurrently with spirometry.

Baseline clinical characteristics according to modified BPD grade are shown in Table 1. Infants with grade III BPD had significantly lower gestational age and birth weight than those with grade I or grade II BPD (both *P* < 0.001). The durations of invasive mechanical ventilation and hospitalization were significantly longer in the grade III group than in the other two groups (both *P* < 0.001). The grade III group also had higher rates of RDS, surfactant therapy, and nosocomial infection than the grade I group (all *P* < 0.05). The 5 min Apgar score was significantly lower in the grade III group than in the grade I group (*P* < 0.01). CRIB-II scores were unavailable.

**Table 1:** Baseline and neonatal characteristics according to BPD severity.

Characteristic	Grade I (*n* = 29)	Grade II (*n* = 23)	Grade III (*n* = 16)	F/chi-square	*P* value
Male sex, *n* (%)	17 (58.6)	14 (60.9)	11 (68.8)	0.477	0.788
Gestational age, wk	30.5 ± 1.3	29.0 ± 1.5	26.8 ± 1.4	35.48	<0.001
Birth weight, g	1,342 ± 198	1,156 ± 234	968 ± 186	16.24	<0.001
Cesarean delivery, *n* (%)	16 (55.2)	13 (56.5)	9 (56.3)	0.011	0.995
Antenatal corticosteroids, *n* (%)	21 (72.4)	16 (69.6)	11 (68.8)	0.083	0.959
5 min Apgar score	8.3 ± 0.9	7.8 ± 1.1	7.0 ± 1.3	7.12	0.002
RDS, *n* (%)	16 (55.2)	18 (78.3)	15 (93.8)	7.414	0.025
Surfactant therapy, *n* (%)	14 (48.3)	16 (69.6)	14 (87.5)	7.138	0.028
PDA, *n* (%)	9 (31.0)	11 (47.8)	10 (62.5)	4.076	0.130
Invasive ventilation, d	3.6 ± 3.2	9.8 ± 7.4	24.6 ± 12.1	38.52	<0.001
Hospital stay, d	42.3 ± 11.6	56.8 ± 15.2	78.4 ± 18.5	29.06	<0.001
IVH grade ≥II, *n* (%)	4 (13.8)	5 (21.7)	6 (37.5)	3.473	0.176
ROP, *n* (%)	5 (17.2)	7 (30.4)	8 (50.0)	5.392	0.067
Nosocomial infection, *n* (%)	14 (48.3)	15 (65.2)	14 (87.5)	6.778	0.034

BPD, bronchopulmonary dysplasia; IVH, intraventricular hemorrhage; PDA, patent ductus arteriosus; RDS, respiratory distress syndrome; ROP, retinopathy of prematurity. Continuous variables are presented as mean ± standard deviation.

### Pulmonary function at 3 years of age

Among the 68 children with BPD, 60 (88.2%) completed the 3-year follow-up visit and valid pulmonary function testing. Eight children were lost to follow-up, including 4 in the grade I group, 3 in the grade II group, and 1 in the grade III group. Reasons for loss to follow-up included relocation (*n* = 5) and parental refusal to continue participation (*n* = 3). Follow-up records did not document severe neurodevelopmental impairment as the reason for missing pulmonary function data. No death was recorded among enrolled children between BPD classification and the 3-year follow-up assessment. Gestational age, birth weight, and modified BPD grade distribution did not differ significantly between children who completed follow-up and those lost to follow-up (all *P* > 0.05).

**Figure 1:**
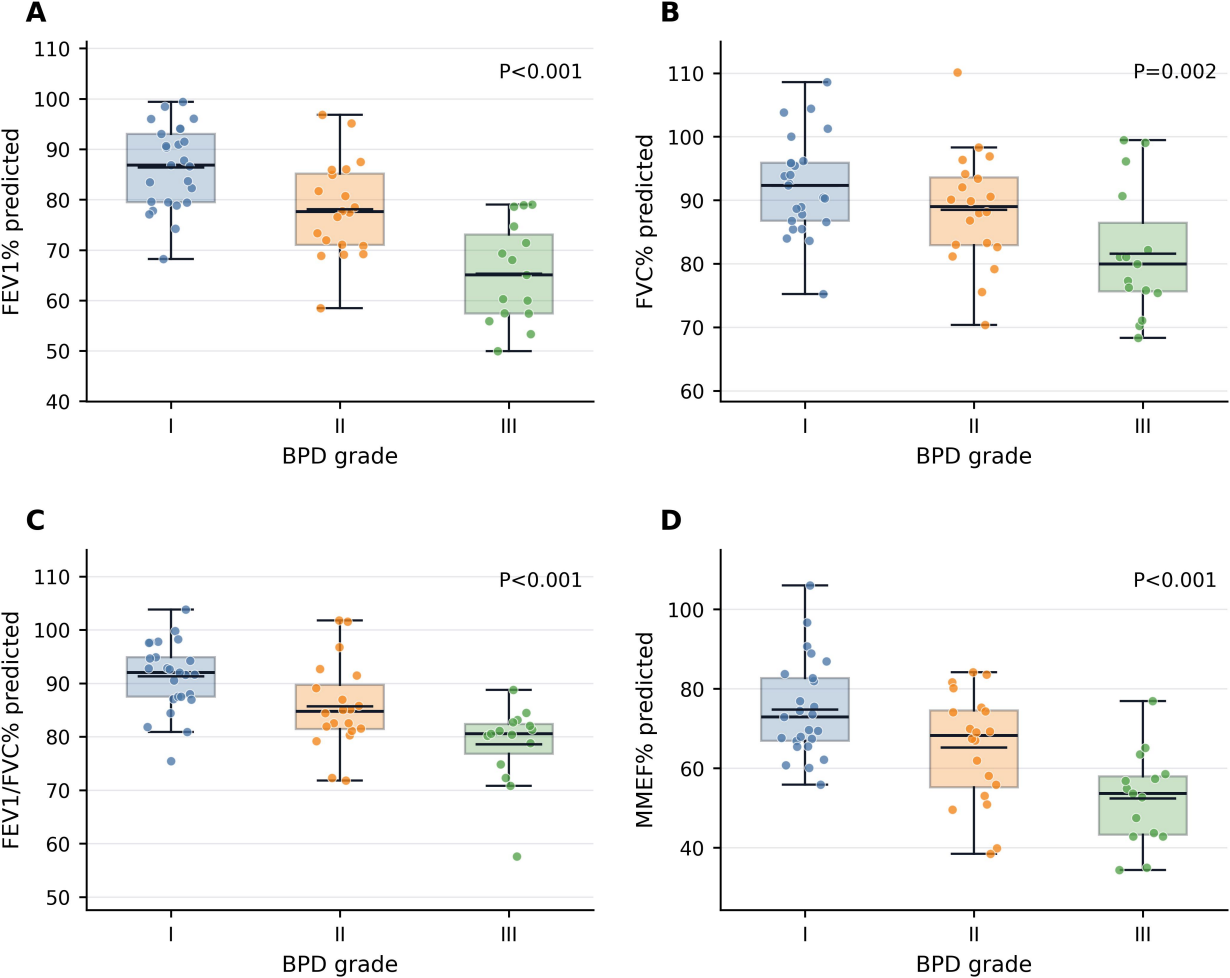
Comparison of main pulmonary function indices at 3 years of age according to BPD severity. Box-and-whisker plots show the distributions of FEV1% predicted, FVC% predicted, FEV1/FVC% predicted, and MMEF% predicted across BPD grade I, II, and III groups. Median pulmonary function values progressively decreased from grade I to grade III, indicating a graded relationship between BPD severity and pulmonary function impairment. The grade III group showed particularly low MMEF% predicted, suggesting prominent small-airway dysfunction.

Pulmonary function indices at 3 years according to BPD grade are presented in Table 2 and Figure 1. Overall, FEV1% predicted, FEV1/FVC% predicted, and MMEF% predicted were below expected normal values across BPD severity groups, indicating variable degrees of pulmonary function impairment. The grade III group had significantly lower FEV1% predicted (65.3 ± 9.8%), FEV1/FVC% predicted (78.6 ± 7.4%), and MMEF% predicted (52.4 ± 11.6%) than the grade I group (86.4 ± 8.2%, 91.3 ± 6.5%, and 74.8 ± 12.3%, respectively) and the grade II group (78.1 ± 9.5%, 85.7 ± 8.1%, and 65.2 ± 13.8%, respectively) (all *P* < 0.001). FVC% predicted also differed significantly among groups (*P* = 0.002). Pairwise comparisons showed significant differences among all three grades for FEV1% predicted and FEV1/FVC% predicted (all *P* < 0.05). For MMEF% predicted, significant differences were observed between grade I and grade III and between grade II and grade III (both *P* < 0.01).

**Table 2:** Pulmonary function indices at 3 years of age according to the BPD severity.

Pulmonary function index	Grade I (*n* = 25)	Grade II (*n* = 20)	Grade III (*n* = 15)	*F* value	*P* value
FEV1% predicted	86.4 ± 8.2	78.1 ± 9.5	65.3 ± 9.8	24.68	<0.001
FVC% predicted	92.3 ± 7.6	88.5 ± 8.9	81.6 ± 10.2	6.93	0.002
FEV1/FVC% predicted	91.3 ± 6.5	85.7 ± 8.1	78.6 ± 7.4	14.56	<0.001
MMEF% predicted	74.8 ± 12.3	65.2 ± 13.8	52.4 ± 11.6	13.87	<0.001

FEV1, forced expiratory volume in 1 s; FVC, forced vital capacity; MMEF, maximal mid-expiratory flow. Values are presented as mea*n* ± standard deviation.

Among the 60 children who completed pulmonary function testing, 34 (56.7%) had obstructive ventilatory impairment, 8 (13.3%) had restrictive ventilatory impairment, 6 (10.0%) had mixed ventilatory impairment, and 12 (20.0%) had normal pulmonary function. The overall rate of abnormal pulmonary function was significantly higher in the grade III group (100.0%, 15/15) than in the grade I group (60.0%, 15/25) and grade II group (80.0%, 16/20) (chi-square = 8.675, *P* = 0.013). In the grade III group, obstructive impairment was the predominant pattern (60.0%, 9/15), followed by mixed impairment (26.7%, 4/15) and restrictive impairment (13.3%, 2/15).

### Asthma diagnosis and clinical risk classification at 3 years of age

**Figure 2:**
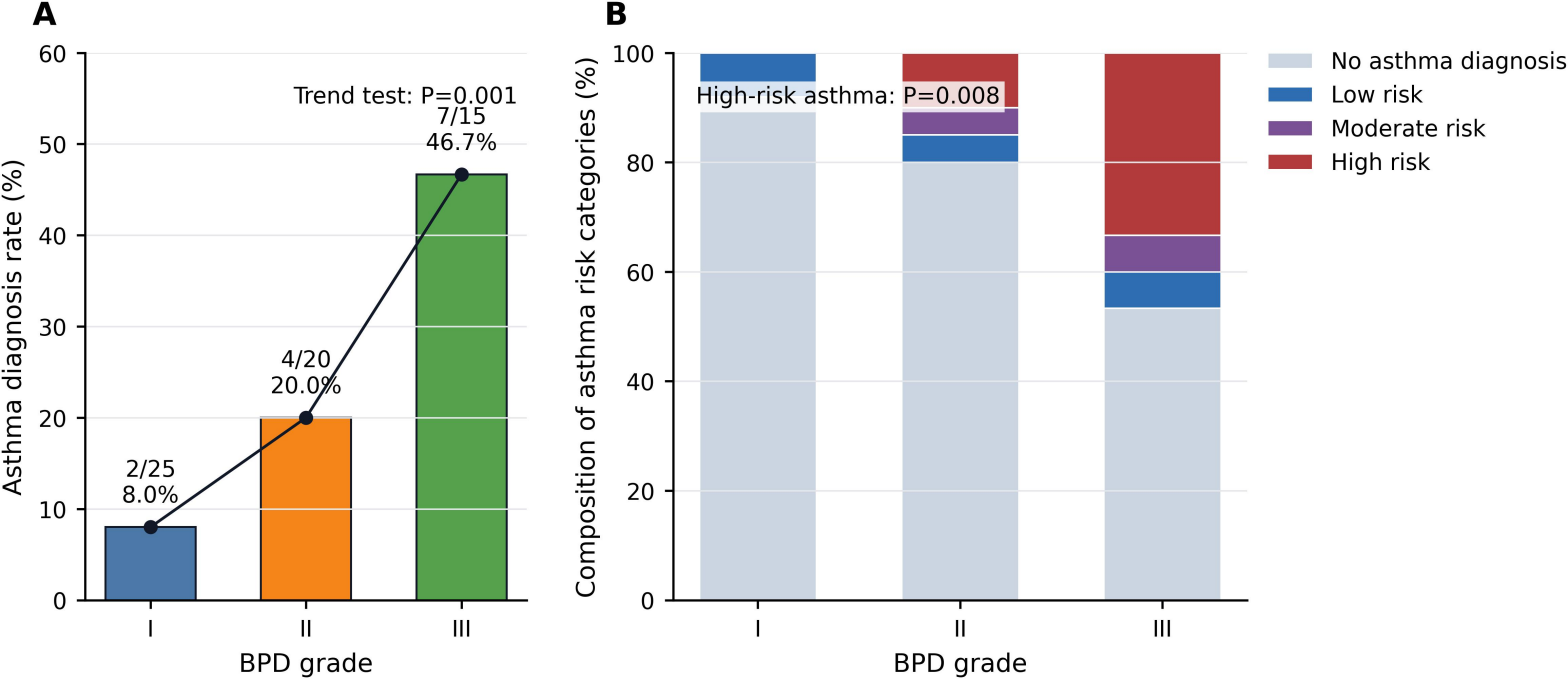
Asthma diagnosis rate and clinical risk categories at 3 years of age according to the BPD severity. The asthma diagnosis rates were 8.0%, 20.0%, and 46.7% in the grade I, grade II, and grade III groups, respectively. The Cochran-Armitage trend test demonstrated a significant increasing trend across BPD severity grades (Z = 3.214, *P* = 0.001). High-risk asthma was most frequent in the grade III group.

Among the 60 children who completed follow-up, 13 (21.7%) were diagnosed with asthma at 3 years of age. The asthma diagnosis rate increased across the BPD severity categories: 2 of 25 children (8.0%) in the grade I group, 4 of 20 (20.0%) in the grade II group, and 7 of 15 (46.7%) in the grade III group had diagnosed asthma. The between-group difference was statistically significant (chi-square = 8.156, *P* = 0.017). The Cochran-Armitage trend test demonstrated a significant increasing trend in asthma diagnosis rate across BPD severity grades (Z = 3.214, *P* = 0.001) (Figure 2).

Asthma risk categories are shown in Table 3. The proportion of children with high-risk asthma was significantly higher in the grade III group (33.3%, 5/15) than in the grade I group (0%) and grade II group (10.0%, 2/20) (Fisher exact test, *P* = 0.008). Among the 13 children diagnosed with asthma, 7 (53.8%) were classified as high risk, indicating that children with BPD who developed asthma tended to require more intensive controller therapy or had more frequent exacerbations.

**Table 3:** Asthma diagnosis and clinical risk categories at 3 years of age according to BPD severity, *n* (%).

Asthma risk category	Grade I (*n* = 25)	Grade II (*n* = 20)	Grade III (*n* = 15)	Total (*n* = 60)
Diagnosed asthma	2 (8.0)	4 (20.0)	7 (46.7)	13 (21.7)
Low risk	2 (8.0)	1 (5.0)	1 (6.7)	4 (6.7)
Moderate risk	0 (0)	1 (5.0)	1 (6.7)	2 (3.3)
High risk	0 (0)	2 (10.0)	5 (33.3)	7 (11.7)
No asthma diagnosis	23 (92.0)	16 (80.0)	8 (53.3)	47 (78.3)

Risk categories were defined according to clinical features and treatment requirements. BPD, bronchopulmonary dysplasia.

### Correlation between BPD grade and pulmonary function indices

**Figure 3:**
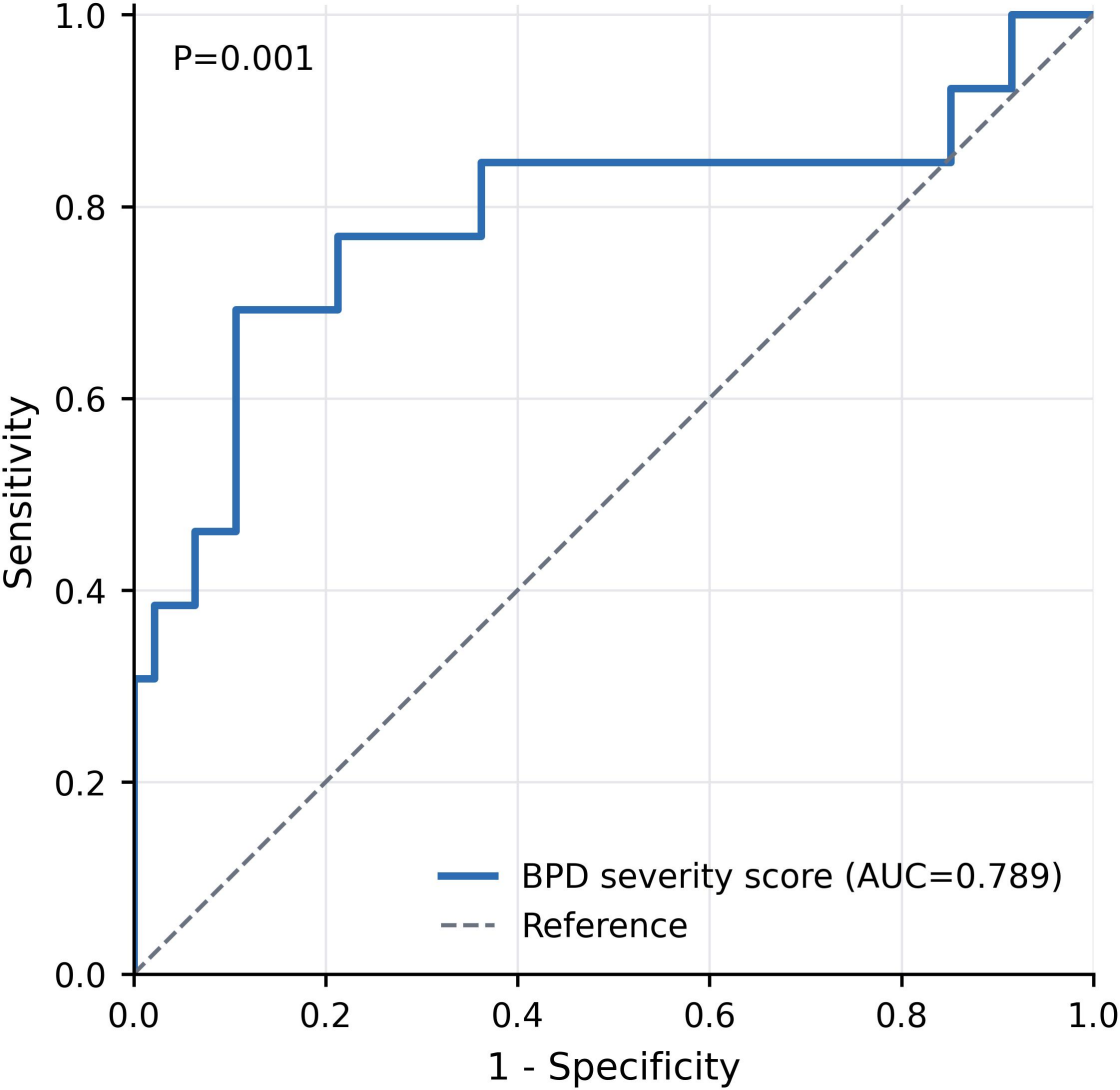
Receiver operating characteristic curve for modified BPD grade in predicting asthma diagnosis at 3 years of age. Modified BPD grade showed moderate-to-good discrimination for asthma diagnosis, with an area under the curve of 0.789 (95% CI, 0.668-0.910; *P* = 0.001). A cutoff of grade II or higher yielded a sensitivity of 84.6% and specificity of 61.7%, whereas a cutoff of grade III yielded a sensitivity of 53.8% and specificity of 88.3%.

Spearman rank correlation analysis showed significant negative correlations between BPD severity grade and FEV1% predicted (*r* = −0.682, *P* < 0.001), FEV1/FVC% predicted (*r* = −0.594, *P* < 0.001), and MMEF% predicted (*r* = −0.613, *P* < 0.001) (Table 4). Modified BPD grade was also moderately and negatively correlated with FVC% predicted (*r* = −0.386, *P* = 0.002) (Figure 3). These findings indicate that higher BPD severity was associated with lower pulmonary function at 3 years of age, with the strongest correlations observed for small-airway function and obstructive indices.

**Table 4:** Spearman correlations between BPD severity grade and pulmonary function indices at 3 years of age.

Pulmonary function index	Spearman *r*	*P* value
FEV1% predicted	−0.682	<0.001
FVC% predicted	−0.386	0.002
FEV1/FVC% predicted	−0.594	<0.001
MMEF% predicted	−0.613	<0.001

BPD, bronchopulmonary dysplasia; FEV1, forced expiratory volume in 1 s; FVC, forced vital capacity; MMEF, maximal mid-expiratory flow.

### Logistic regression analysis of factors associated with asthma diagnosis at 3 years of age

Asthma diagnosis at 3 years of age was used as the dependent variable. Candidate independent variables included modified BPD grade (with grade I as the reference category), gestational age, birth weight <1,000 g, duration of invasive mechanical ventilation, RDS, PDA, and nosocomial infection. Variables with *P* < 0.10 in univariable logistic regression, including modified BPD grade, birth weight <1,000 g, duration of invasive mechanical ventilation, and nosocomial infection, were entered into the multivariable logistic regression model.

**Figure 4:**
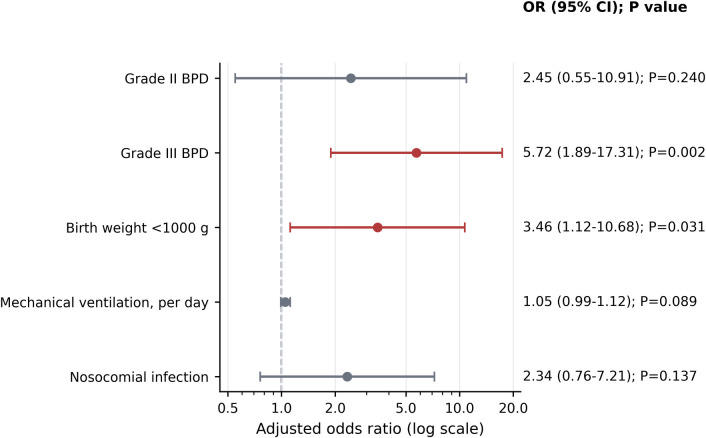
Multivariable logistic regression forest plot for factors associated with asthma diagnosis at 3 years of age. The forest plot summarizes adjusted odds ratios and 95% confidence intervals from the exploratory multivariable logistic regression model. Grade III BPD within the modified severity category and birth weight <1,000 g were associated with asthma diagnosis at 3 years of age, whereas duration of mechanical ventilation and nosocomial infection showed positive but statistically nonsignificant associations.

In the exploratory multivariable model, grade III BPD (OR, 5.72; 95% CI, 1.89–17.31; *P* = 0.002) and birth weight <1,000 g (OR, 3.46; 95% CI, 1.12–10.68; *P* = 0.031) were associated with asthma diagnosis at 3 years of age (Table 5 and Figure 4). Duration of invasive mechanical ventilation, analyzed per 1-day increase, showed a positive but statistically nonsignificant association (OR, 1.05; 95% CI, 0.99–1.12; *P* = 0.089). Nosocomial infection also showed a positive but statistically nonsignificant association (OR, 2.34; 95% CI, 0.76–7.21; *P* = 0.137).

**Table 5:** Multivariable logistic regression analysis of factors associated with asthma diagnosis at 3 years of age.

Variable	Beta	SE	Wald chi-square	OR (95% CI)	*P* value
BPD grade (grade I as reference)			6.483		0.039
Grade II BPD	0.896	0.762	1.382	2.45 (0.55–10.91)	0.240
Grade III BPD	1.743	0.566	9.487	5.72 (1.89–17.31)	0.002
Birth weight <1,000 g	1.242	0.575	4.660	3.46 (1.12–10.68)	0.031
Duration of mechanical ventilation, per day	0.052	0.031	2.886	1.05 (0.99–1.12)	0.089
Nosocomial infection	0.851	0.574	2.197	2.34 (0.76–7.21)	0.137

BPD, bronchopulmonary dysplasia; CI, confidence interval; OR, odds ratio; SE, standard error. The model was exploratory because 13 asthma events were available.

## Discussion

In this prospective cohort study, we systematically evaluated pulmonary function and asthma diagnosis risk at 3 years of age among preterm children with BPD identified using a prespecified operational definition and classified using a modified severity scheme based on respiratory support and oxygen requirement. The principal findings were as follows. First, modified BPD grade was significantly and negatively associated with FEV1% predicted, FEV1/FVC% predicted, and MMEF% predicted, indicating that more severe neonatal BPD within this scheme was associated with greater preschool pulmonary function impairment. Second, children with grade III BPD had an asthma diagnosis rate of 46.7% at 3 years of age, substantially higher than the rates in the grade I and grade II groups. Third, grade III BPD and birth weight <1,000 g were associated with asthma diagnosis in an exploratory adjusted model. These results support the use of this BPD severity category as a clinical risk marker, but they do not establish that modified BPD grade is causally independent of the broader effects of extreme prematurity.

### Effect of BPD severity on long-term pulmonary function

In the present cohort, the mean FEV1% predicted at 3 years of age was 65.3% in the grade III BPD group, significantly lower than in the grade I and grade II groups. Moreover, all children with grade III BPD had some form of pulmonary function abnormality. These findings are consistent with international longitudinal studies showing persistent reductions in lung function among extremely preterm survivors, especially those with BPD (16–22). For example, the EPICure cohort demonstrated substantially lower FEV1 in children born extremely preterm at 11 years of age, and BPD severity was associated with greater impairment (21). Longitudinal data from childhood to adulthood have similarly shown persistently reduced lung function among individuals born preterm with BPD (22).

An important observation in this study was that MMEF% predicted was markedly reduced, particularly in the grade III group. MMEF is effort-dependent and should not be interpreted in isolation, but a consistent reduction alongside lower FEV1/FVC supports the presence of persistent small-airway dysfunction. This pattern is consistent with contemporary concepts of prematurity-associated lung disease, in which respiratory morbidity after very preterm birth reflects interacting airway, parenchymal, vascular, and developmental components rather than traditional BPD alone. It is also biologically plausible because impaired alveolar septation, reduced gas-exchange surface area, dysregulated microvascular development, and airway remodeling can reduce pulmonary functional reserve and contribute to persistent airflow limitation (1–4, 20, 23, 36–38).

The distribution of ventilatory impairment also deserves attention. Obstructive impairment was the predominant pattern in the grade III group, but a substantial proportion of children had mixed impairment. This pattern supports the concept that BPD-related respiratory morbidity is not limited to classic obstructive airway disease. Instead, it reflects the combined consequences of abnormal airway growth, parenchymal simplification, altered lung mechanics, and, in some patients, pulmonary vascular disease that may influence later respiratory outcomes (2, 18–20, 39).

### Predictive value of BPD severity for asthma diagnosis risk

The asthma diagnosis rate at 3 years of age was 46.7% in children with grade III BPD. In addition, most children with diagnosed asthma were classified as high risk, suggesting a greater requirement for controller therapy or a higher exacerbation burden. In the exploratory adjusted model, grade III BPD remained associated with asthma diagnosis, with an OR of 5.72. This finding suggests that severe BPD may identify a clinically important subgroup of preterm children who require intensified respiratory surveillance during the preschool period.

The association between BPD and later asthma or asthma-like morbidity has been reported in previous studies and meta-analyses (24–26, 36, 40). A recent systematic review and meta-analysis found that preterm infants with BPD had a higher risk of asthma later in life (24). Population-based and cohort studies have also reported increased wheezing disorders, lower respiratory tract infections, and asthma diagnoses among children with BPD during early childhood (26, 36, 37, 40). The effect size observed in the present study for grade III BPD was higher than pooled estimates reported for BPD overall. This may reflect the modified support- and oxygen-based severity categorization used here, the preschool age at outcome assessment, and the greater clinical vulnerability of infants with severe neonatal lung disease. However, it may also reflect residual confounding and collinearity among BPD severity, gestational age, birth weight, duration of respiratory support, infection, and other consequences of extreme prematurity. For this reason, severe BPD should be interpreted as an integrated clinical marker of early lung injury and prematurity-related vulnerability rather than a fully separable causal exposure.

From a pathophysiologic perspective, post-BPD asthma-like disease may reflect multiple overlapping mechanisms. Structural airway narrowing and remodeling after disrupted lung development may cause persistent airflow limitation. Chronic inflammation related to prematurity, oxygen exposure, mechanical ventilation, and infection may contribute to airway hyperresponsiveness. In addition, impaired epithelial barrier function and altered immune responses may increase susceptibility to viral infections and allergen sensitization (21–26, 37). However, BPD-related wheeze should not be regarded as identical to classic allergic asthma, especially at 3 years of age. The former may be dominated by structural post-prematurity airway disease and may respond variably to bronchodilators or ICS, whereas classic allergic asthma is more strongly characterized by reversible airflow limitation and type 2 airway inflammation. Therefore, clinical evaluation should integrate symptom patterns, bronchodilator response, biomarkers when available, atopy history, and differential diagnosis before long-term controller therapy is escalated.

### Clinical implications

The findings of this study have several implications for clinical practice. First, the BPD severity category at 36 weeks' postmenstrual age may be useful for risk stratification beyond the neonatal hospitalization in similar follow-up settings. Children with grade III BPD should be considered a higher-risk group for preschool pulmonary function impairment and asthma-like morbidity. Structured follow-up should include recurrent symptom assessment, infection prevention counseling, evaluation of exercise tolerance, and objective lung function monitoring when feasible.

Second, clinicians should be cautious when interpreting recurrent wheeze in children with a history of BPD. Because BPD-related airway disease and preschool asthma overlap clinically, a multidisciplinary approach involving neonatology, pediatric pulmonology, and primary care may improve diagnostic accuracy and treatment decisions. Where feasible, bronchodilator testing, fractional exhaled nitric oxide testing, allergy assessment, and careful evaluation of treatment response may help distinguish inflammatory asthma phenotypes from structural post-prematurity respiratory disease (27–35).

Third, birth weight <1,000 g was associated with asthma diagnosis at 3 years of age in the exploratory adjusted model. This finding supports the need for integrated risk assessment incorporating both BPD severity and perinatal vulnerability. Infants with extremely low birth weight and moderate-to-severe BPD may benefit from more intensive respiratory follow-up and earlier intervention.

Finally, the present study provides real-world evidence from a Chinese single-center cohort supporting the prognostic value of a prespecified modified BPD severity category. Although the sample size was limited, the graded associations observed across pulmonary function, asthma diagnosis, and asthma risk categories suggest that this neonatal severity classification may help identify children requiring individualized long-term respiratory care. This interpretation should remain tied to the operational definition and modified categorization used in this cohort.

## Limitations

This study has several limitations. First, it was a single-center cohort study with a relatively small sample size, which may limit generalizability and reduce statistical power for subgroup analyses. Only 13 asthma events occurred, so the multivariable logistic regression model was exploratory and may be unstable. Penalized regression or alternative sensitivity models were not performed in this dataset; therefore, larger validation cohorts are needed before the adjusted effect estimates can be considered robust. CRIB-II scores were unavailable, which limited our ability to adjust for or describe global neonatal illness severity using a validated composite score.

Second, the cohort was conditional on survival to BPD assessment at 36 weeks' postmenstrual age and on participation in a structured follow-up program. No deaths occurred among enrolled children after BPD classification, and follow-up records did not identify severe neurodevelopmental impairment as a reason for missing pulmonary function testing. These findings are notable compared with published cohorts of very preterm infants with moderate-to-severe BPD. They may reflect the small cohort size, single-center case mix, exclusion of major congenital disorders, survival-to-classification selection, local neonatal care pathways, and close family engagement in the follow-up program. They should not be interpreted as evidence that mortality or neurodevelopmental morbidity is low among all infants with BPD.

Third, the relatively high spirometry completion rate at 3 years may reflect the selected follow-up population, repeated outpatient contact, and testing by trained pediatric pulmonary function technicians. Formal Bayley-III cognitive or motor testing was not performed concurrently with spirometry, so milder neurodevelopmental impairment may have been under-recognized. If children with more severe neurodevelopmental or respiratory impairment were less likely to attend follow-up or complete valid spirometry, the severity gradient in pulmonary function may have been underestimated.

Fourth, external generalizability outside China and outside similar single-center follow-up systems should be considered limited. Neonatal respiratory management, discharge thresholds, family adherence to follow-up, access to pediatric pulmonary function testing, and preschool asthma diagnostic practices may differ across countries and health systems. The findings therefore require validation in larger multicenter cohorts that include diverse NICU practices and broader post-discharge care pathways.

Fifth, follow-up was limited to 3 years of age. Preschool asthma diagnosis is inherently challenging because wheezing phenotypes may remit or evolve with age; therefore, longer follow-up into school age is needed to confirm the stability of the present findings. Sixth, there was no healthy term-born control group. Pulmonary function interpretation was based on percentages of predicted values, and reference equations for Chinese preschool children require further standardization. Seventh, inflammatory biomarkers such as fractional exhaled nitric oxide, allergic sensitization, and detailed atopy-related family history were not systematically measured, limiting our ability to distinguish BPD-related structural wheeze from allergic asthma phenotypes.

Finally, the operational BPD definition and modified severity categorization require careful interpretation. We defined BPD at 36 weeks' postmenstrual age as persistent supplemental oxygen requirement (FiO2 > 0.21) to maintain target oxygen saturation of 90%–95%, together with persistent pulmonary parenchymal abnormalities on chest imaging. We then categorized severity according to respiratory support mode and FiO2 thresholds. This prespecified approach was used because these variables were complete in the local electronic medical record, whereas some flow-based support details required for exact NICHD or Jensen classification were incomplete. The scheme should not be interpreted as a new general BPD definition or as interchangeable with published NICHD or Jensen definitions. The findings should therefore be validated in larger multicenter cohorts using internationally standardized BPD definitions.

## Conclusions

Using a prespecified operational BPD definition and modified support- and oxygen-based severity categorization, higher neonatal modified BPD grade was associated with pulmonary function impairment at 3 years of age. Higher modified BPD grade was associated with lower FEV1, FEV1/FVC, and MMEF and with a higher prevalence of ventilatory impairment, with small-airway dysfunction being particularly prominent.

Children with grade III BPD had a substantially increased asthma diagnosis rate at 3 years of age, and grade III BPD remained associated with asthma diagnosis in an exploratory adjusted model. Birth weight <1,000 g was another associated risk marker. These findings support cautious incorporation of this modified BPD severity category and extremely low birth weight into local long-term respiratory risk stratification for preterm children, while recognizing that preschool asthma diagnosis overlaps clinically with post-prematurity airway disease.

Children with severe BPD should receive structured long-term follow-up that includes pulmonary function monitoring, asthma-like symptom surveillance, respiratory infection prevention, and individualized respiratory management. Multicenter studies with larger sample sizes, longer follow-up into school age, and standardized NICHD or Jensen BPD definitions are needed to validate these findings and refine post-BPD respiratory care pathways.

## Data Availability

The raw data supporting the conclusions of this article will be made available by the authors, without undue reservation.
